# The double-faced role of P2X_7_ receptors in toxin-induced animal models of Parkinson’s disease

**DOI:** 10.1186/1471-2210-11-S2-A56

**Published:** 2011-09-05

**Authors:** Beáta Sperlágh, Zsuzsanna Hracskó, Mária Baranyi, Cecília Csölle, Flóra Gölöncsér, Ágnes Kittel, Emilia Madarász

**Affiliations:** 1 Department of Pharmacology, Institute of Experimental Medicine, Hungarian Academy of Sciences, 1083 Budapest, Hungary; 2 Department of Gene Technology and Developmental Neurobiology, Institute of Experimental Medicine, Hungarian Academy of Sciences, 1083 Budapest, Hungary

## Background

Previous studies indicate a role of P2X_7_ receptors in processes that lead to neuronal death. The main objective of our study was to examine whether genetic deletion or pharmacological blockade of P2X_7_ receptors influenced dopaminergic cell death in various models of Parkinson’s disease (PD).

## Methods

PC12 cells and primary mesencephalic neurons were used in culture, and the striatum and the substantia nigra were prepared from wild-type and P2X_7_ receptor knockout mice. Rotenone and 1-methyl-4-phenyl-1,2,3,6-tetrahydropyridine (MPTP) treatments were applied *in vitro* and *in vivo* to reproduce neurochemical hallmarks of PD. Receptor expression, cell survival indicators, and endogenous biogenic amine, amino acid, adenine nucleotide and endocannabinoid contents were analyzed.

## Results

mRNA encoding P2X_7_ and P2X_4_ receptors was up-regulated after treatment of PC12 cells with MPTP. P2X_7_ antagonists protected against MPTP- and rotenone-induced toxicity in the LDH assay, but failed to protect after rotenone treatment in the MTT assay in PC12 cells and in primary midbrain culture. *In vivo* MPTP and *in vitro* rotenone pretreatments increased the mRNA expression of P2X_7_ receptors in the striatum and substantia nigra of wild-type mice. Basal mRNA expression of P2X_4_ receptors was higher in P2X_7_ knockout mice and was further up-regulated by MPTP treatment. Genetic deletion or pharmacological inhibition of P2X_7_ receptors did not change survival rate or depletion of striatal endogenous dopamine (DA) content after *in vivo* MPTP or *in vitro* rotenone treatment. However, depletion of norepinephrine was significant after MPTP treatment only in P2X_7_ knockout mice. The basal ATP content was higher in the substantia nigra of wild-type mice, but the ADP level was lower. Rotenone treatment elicited a similar reduction in ATP content in the substantia nigra of both genotypes, whereas reduction of ATP was more pronounced after rotenone treatment in striatal slices of P2X_7_-deficient mice. Although the endogenous amino acid content remained unchanged, the level of the endocannabinoid, 2-arachidonoyl glycerol (2-AG), was elevated by rotenone in the striatum of wild-type mice, an effect that was absent in mice deficient in P2X_7_ receptors.

## Conclusions

We conclude that P2X_7_ receptor deficiency or inhibition does not support the survival of dopaminergic neurons in *in vivo* or *in vitro* models of PD.

